# Integrated Optical System for Mobile Atomic Interferometric Gravity Gradient

**DOI:** 10.3390/s26154983

**Published:** 2026-08-06

**Authors:** Jipeng Wang, Bangcheng Han, Jinhai Bai, Yu Wang

**Affiliations:** 1 School of Instrumentation and Optoelectronic Engineering, Beihang University, Beijing 100191, China; by2117160@buaa.edu.cn; 2 AVIC Changcheng Institute of Metrology and Measurement, Beijing 100095, China; baijh003@avic.com (J.B.); wangy158@avic.com (Y.W.)

**Keywords:** atomic interferometry, gravity gradiometry, integrated optical system, low power consumption

## Abstract

**Highlights:**

**What are the main findings?**

- A highly integrated, low-power optical system is developed for mobile atomic interferometric gravity gradient measurement, using only 133 MHz and 400 MHz frequency shifts to simplify the system architecture.
- A high-isolation RF driving module is designed to replace traditional mechanical switches, enabling precise laser control and light leakage suppression.

**What are the implications of the main findings?**

- The integrated optical system achieves an atomic interferometry gravity gradient measurement stability of 1.08 E (1 E = 10^−9^ s^−2^), supporting reliable field-deployable and mobile quantum gravity gradient sensing.
- The switch-free, low-power, and miniaturized design provides a practical technical scheme for the engineering and portable application of atomic interferometry gradiometers.

**Abstract:**

This study presents the development of an integrated optical system suitable for mobile atomic interferometry-based gravity gradient measurements. The system exhibits distinct technical advantages, including a simplified frequency-shifting scheme, low laser power consumption, and elimination of mechanical switches. In terms of frequency-shifting design, gravity gradient measurements via atomic interferometry are accomplished using only two frequency shifts at 133 MHz and 400 MHz, which effectively streamlines the optical configuration and significantly reduces the complexity of the supporting radio-frequency (RF) driving system. For power consumption control, a dual-channel fiber laser architecture is adopted, where the total laser power required for atomic interferometry gravity gradient measurements is satisfied with relatively low output power. Furthermore, to meet the laser isolation requirements during measurements, a high-isolation RF driving module is developed, replacing conventional mechanical switches and enabling the integration and miniaturization of the optical system. Using this integrated optical setup, mobile gravity gradient measurement experiments based on atomic interferometry are performed, achieving high-precision mobile atomic interferometric gravity gradient measurement with a long-term stability of 1.08 E (1 E = 10^−9^ s^−2^).

## 1. Introduction

Atomic interferometric gravity gradient measurement technology, featuring ultra-high measurement accuracy and excellent spatial resolution, has emerged as a highly promising research method for geophysical exploration, deep resource prospecting, and the test of fundamental physical constants [1,2,3]. Breakthroughs in its measurement performance hold significant scientific and engineering value for the development of related fields. As the core control unit of atomic interferometric gravity gradient measurement, the laser system serves as a key carrier for atomic cooling, initial state preparation, interferometric manipulation, and detection. Its structural design and performance specifications directly determine the stability of the atomic interference process and the final accuracy of gravity gradient measurement [4,5,6,7]. Therefore, the development of a dedicated high-performance laser system tailored to the requirements of atomic interferometric gravity gradient measurement constitutes a core step for the practical implementation of this technology.

At present, the optical path architecture employed in atomic interferometry gravity gradient experiments adopts a multi-channel independent frequency-shifting framework to accommodate the frequency requirements of lasers for different functions. Given that the operating frequencies of cooling, repump, probe, Raman, and blow lasers in atomic interferometry experiments are mutually independent, conventional schemes rely on master–slave laser frequency-locking references, requiring dedicated frequency-shifting optical paths and supporting radio-frequency (RF) drive circuits for each laser, as well as multiple beam splitting operations to realize frequency division multiplexing of multi-channel lasers [8,9,10]. Although this architecture can satisfy the frequency matching and temporal control demands of multi-mode lasers, it suffers from distinct structural drawbacks that severely restrict the advancement of instruments toward miniaturization, low power consumption, high stability, and field-deployable mobile applications.

In terms of laser power utilization, conventional architectures based on multi-stage beam splitting and multi-channel frequency shifting introduce considerable power loss. The inherent loss of optical beam-splitting components can exceed 10%, and a single frequency-shifting operation using an acousto-optic modulator (AOM) typically induces a power loss of approximately 10% [11,12,13]. Moreover, the double-pass optical path, which is essential in gravity gradient experiments, further exacerbates power attenuation. After multi-stage optical path cascading, the effective laser power efficiency is significantly degraded. To compensate for such optical losses and ensure sufficient laser power for atomic cooling and interferometric measurement, the system must employ high-power lasers with output power of 1 W or higher. This not only substantially increases the overall operating power consumption, conflicting with the low-power design requirements of portable field instruments, but also leads to continuous degradation of core optical components—such as optical windows, lenses, and mirrors—under long-term high-power laser irradiation. Consequently, component aging is accelerated, compromising the long-term measurement stability and service life of the instrument.

In terms of system integration and timing control, the multi-channel independent frequency-shifting optical path is equipped with multiple radio-frequency (RF) driving modules, resulting in high redundancy and complex circuit and optical architectures, which greatly increase system debugging difficulty and integration cost [14]. Meanwhile, to meet the stringent timing requirements of an ultra-high laser extinction ratio in atomic interferometry experiments, conventional schemes generally adopt mechanical switches for fast switching of cooling, repump, and probe lasers. Mechanical switches not only occupy considerable space for optical integration and restrict the miniaturization of the instrument but also require independent timing control programs to achieve precise synchronization with the interferometric system, leading to complicated and cumbersome control logic. More critically, mechanical structures suffer from inherent physical wear, and their failure rate increases significantly under field mobile testing and repeated handling. This can easily induce laser switching failure and timing disorders, severely degrading the repeatability and stability of gravity gradient measurements and greatly limiting the engineering implementation and routine field deployment of atomic interferometry gravity gradiometers.

In response to a series of limitations in conventional atomic interferometry optical systems, including low laser power efficiency, complicated frequency-shifting architecture, high hardware redundancy, poor stability of mechanical switches, and weak field adaptability, this study proposes an integrated laser optical system featuring low power consumption, simplified configuration, laser function multiplexing, and elimination of mechanical switches. By optimizing the optical frequency-shifting architecture and discarding the redundant design of multi-channel independent frequency shifting, the optical layout and radio-frequency (RF) driving modules are considerably simplified, and multi-stage laser power losses are effectively reduced. Meanwhile, an optical timing modulation scheme is employed to replace traditional mechanical switches, which streamlines the hardware structure and eliminates potential mechanical failures while maintaining an ultra-high laser extinction ratio. This design effectively lowers the required laser output power, reduces the overall power consumption and volume, and improves the system timing stability and environmental adaptability, providing a novel optical solution for miniaturization, low-power operation, and highly reliable field engineering applications of atomic interferometry gravity gradiometers.

## 2. Materials and Methods

The design of the optical system for an atomic interferometry gravity gradiometer is critically determined by the appropriate selection of the laser frequency locking point. As the key starting point of the system design, it directly defines the optical path configuration and core component selection, forming the foundation and prerequisite of the entire optical scheme. In this paper, the master laser is locked to the rubidium atomic saturation absorption spectrum to maintain a stable laser frequency. After the master laser output is frequency-shifted by an acousto-optic modulator, a repump laser and a primary Raman laser are generated separately. The slave laser is locked to the master laser via an optical phase-locked loop system, and its output frequency can be flexibly tuned by adjusting the frequency of the phase-locked reference signal source. This tuning not only matches the laser frequencies required for the cooling beam and polarization gradient cooling but also satisfies the frequency chirp requirements of the Raman laser. In conventional schemes, the Raman laser is typically red-detuned relative to the resonant frequency from the ground state to the excited state F′ = 0, and the frequency difference between the Raman laser and the cooling (or repump) laser is approximately 1 GHz. This configuration not only complicates the Raman laser preparation process but also causes significant laser power loss. To address this issue, a blue-detuned Raman beam scheme is proposed in this work, where the Raman beam frequency is set to a blue-detuned position relative to the resonant frequency from the ground state to the excited state F′ = 3, thereby simplifying the overall optical path design.

Modulation transfer spectroscopy (MTS) is utilized for laser frequency stabilization [15,16,17]. As the locking point of the master laser (L1) directly determines the frequency shifting scheme of the subsequent optical path, the detailed frequency-locking configuration is designed as follows. The master laser (L1) is modulated by a fiber-integrated electro-optic modulator with an arbitrarily adjustable modulation frequency. The generated −1st-order sideband is locked to the resonance peak of the 85Rb D2 line corresponding to the transition from F = 3 to F′ = 4 via the modulation transfer stabilization technique. In this case, the 0th-order sideband, namely the output frequency of the master laser (L1), is blue-detuned relative to the resonant transition frequency of the 87Rb D2 line from F = 1 to F′ = 2. The slave laser (L2) is phase-locked to the master laser through an optical phase-locked loop referenced to a standard signal source, and its output frequency can be conveniently varied by adjusting the frequency of the reference source.

### 2.1. Laser Frequency Design Scheme

**Figure 1:**
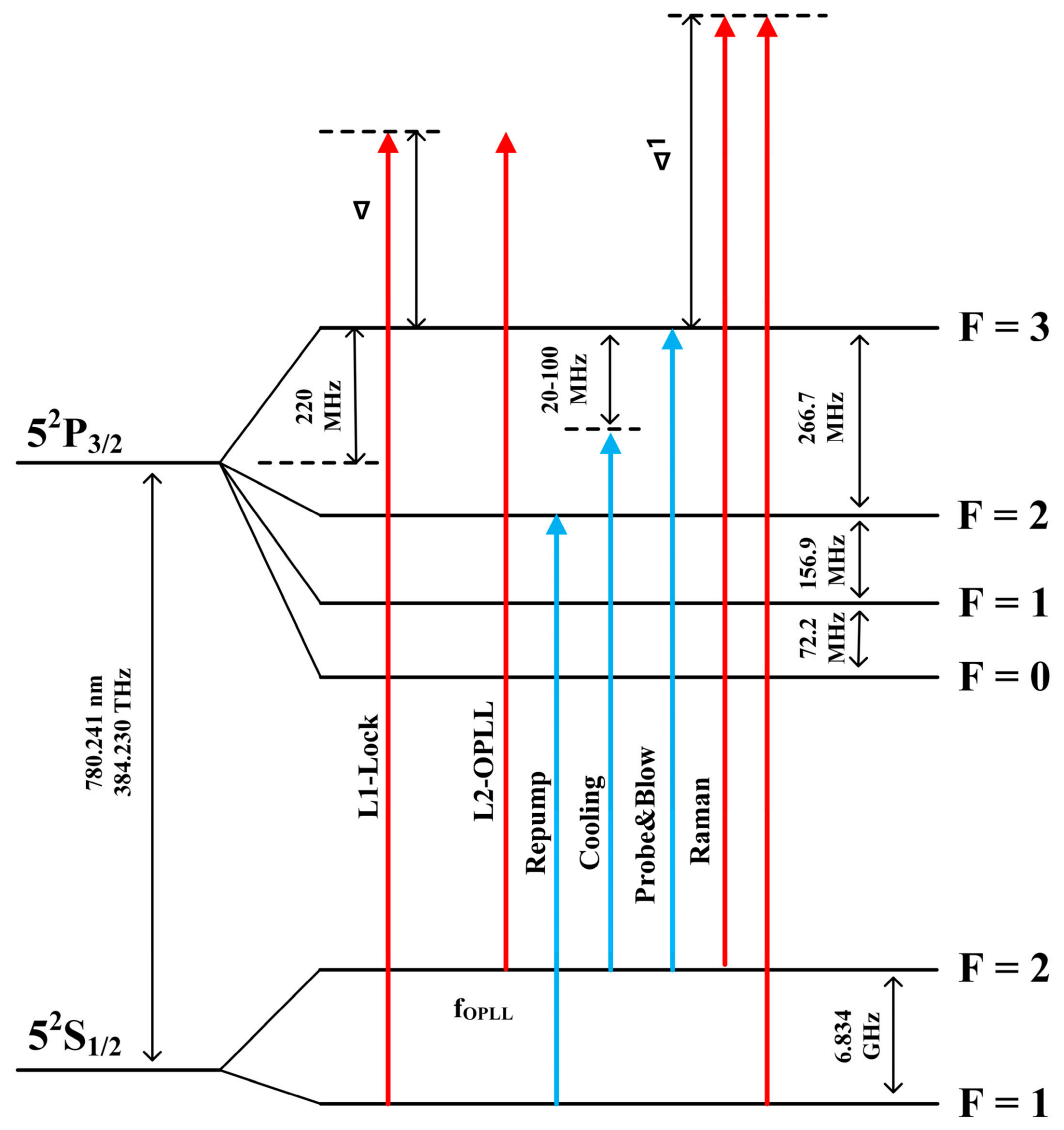
Laser frequency lock and frequency shift scheme design.

Figure 1 shows the frequency diagram of laser frequency locking and frequency shifting for the atomic gravity gradiometer, which requires multiple laser beams including cooling, repump, probe, blow, and Raman beams. The cooling beam is red-detuned by approximately 20 MHz relative to the resonant transition frequency of the rubidium D2 line from the ground state F = 2 to the excited state F′ = 3, and the detuning is increased to 60–100 MHz during the polarization gradient cooling stage. The repump beam is tuned to the resonant transition frequency from the ground state F = 1 to the excited state F′ = 2, while the push-out laser and probe laser are set to the resonant transition frequency from the ground state F = 2 to the excited state F′ = 3. For the two Raman beams, the slave Raman laser is blue-detuned by a frequency Δ1 relative to the resonant transition frequency from the ground state F = 2 to the excited state F′ = 3.

### 2.2. Dual-Frequency-Shifting Low-Power Optical Path Design

Based on the laser frequency-locking scheme designed in the previous section, we propose a configuration that requires only two distinct frequency shifts and demands significantly lower laser input power compared with existing optical systems.

#### 2.2.1. Frequency-Shifting Optical Path Scheme

In this study, the laser system adopts a dual-channel fiber laser as the source to emit two independent laser beams. After frequency locking via the MTS method, Laser L1 is split by Beam Splitter 1 and serves as the repump laser and one component of the Raman laser. Specifically, the repump laser is generated by frequency-shifting the main laser beam via a 400 MHz acousto-optic modulator (AOM).

The second laser beam L2 is phase-locked via an optical phase-locked loop (OPLL) [18] and configured to generate cooling, probe, blow, and Raman lasers. In atomic interferometry experiments, the cooling, probe, blow, and Raman lasers are activated at distinct time sequences. Taking advantage of the reference radio-frequency signal for beam frequency calibration inside the OPLL system, the output frequency of the L2 laser can be dynamically adjusted at different experimental stages.

After passing through Beam Splitter 2, the L2 laser is divided into two branches. The first branch is combined with the L1 laser to form the Raman laser beam. The merged beam propagates through a 2 × 2 beam splitter, with one split beam serving as the reference laser for OPLL phase locking and the other frequency-shifted by a 400 MHz acousto-optic modulator (AOM) to generate the Raman laser. The second branch of the L2 laser undergoes a 133 MHz AOM frequency shift to produce cooling and blow laser outputs.

Given the asynchronous activation of cooling and probe lasers, the diffractive characteristics of the AOM are fully exploited in this design. The −1st-order diffracted beam of the AOM acts as the cooling laser and blow laser, while the 0th-order transmitted beam serves as the probe laser. During the magneto-optical trap (MOT) atom loading process, frequency tuning of the OPLL cooperates with AOM activation to generate the cooling laser for atom trapping. Upon the completion of MOT loading, OPLL frequency hopping is performed to realize polarization gradient cooling via the cooling laser. After polarization gradient cooling, further OPLL frequency shifting converts the cooling laser into a blow laser for atomic state selection.

Once the state selection procedure is finished, the AOM is switched off to completely terminate the cooling laser output, while the probe laser is activated. When the falling atomic cloud enters the detection region, real-time OPLL frequency hopping is implemented to achieve fluorescence signal detection.

This proposed method leverages the time-sequenced activation of different lasers and the frequency-hopping capability of the OPLL system. It repurposes the unused 0th-order transmitted beam of the high-power cooling laser after MOT loading, which otherwise wastes laser power in conventional schemes. This strategy significantly simplifies optical configuration and effectively reduces the overall power requirement of the laser source.

#### 2.2.2. Optical Power Design Scheme

This design simplifies the overall optical system architecture via optimized frequency-shifting strategies and reconstructs the laser driving and optical transmission modules, yielding a substantial reduction in required laser input power. In contrast to the high-power laser configurations of conventional atomic interferometric gravity gradient measurement systems, the proposed low-power laser scheme offers distinct advantages for mobile field measurements.

First, it reduces total system power consumption, extending the continuous operational duration of the instrument under off-grid field conditions and improving field endurance. Second, lowered power dissipation suppresses thermal generation and mitigates thermally induced drift of critical optical components. Third, the reduced laser output power alleviates irradiation-induced damage and aging of optical elements, protecting core hardware and enhancing long-term system reliability and service lifetime.

Furthermore, the low-power architecture facilitates system miniaturization and integration, satisfying the core demands of mobile gravity gradiometers for lightweight packaging, portability, and environmental robustness. This provides a solid foundation for high-precision, long-term stable field measurement of atomic interferometric gravity gradients.

**Figure 2:**
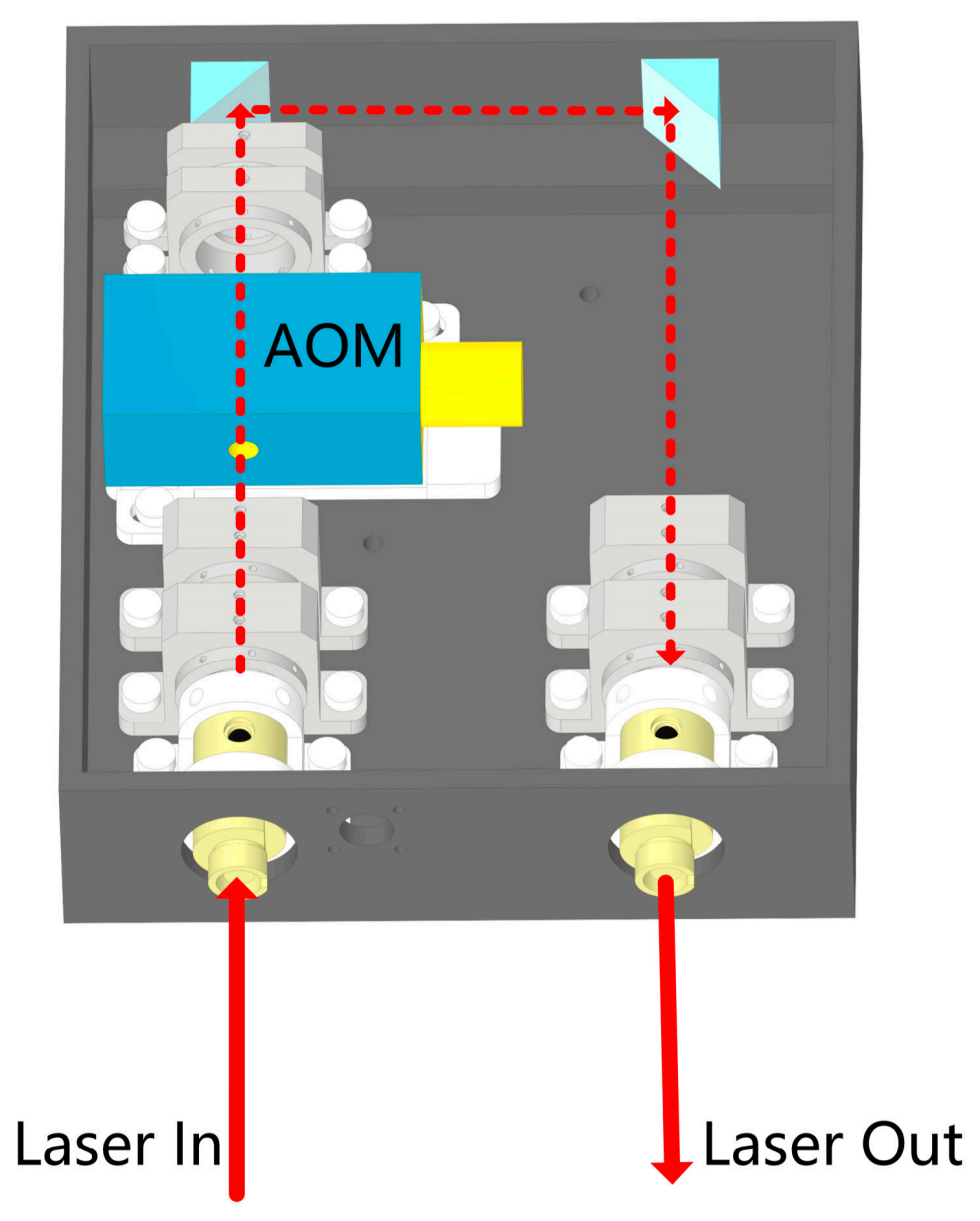
Schematic of the adhesively assembled frequency-shifting optical path.

To maximize laser power utilization, all AOM frequency-shifting optical segments are assembled via adhesive bonding packaging. Through precise optical alignment, the overall optical loss of the system is constrained to approximately 10%. The design scheme of the frequency-shifting module is shown in Figure 2.

To effectively control the loss of the beam-splitting optical path, we adopt an adhesively bonded beam-splitting module. Meanwhile, a calibrated fiber collimator array is utilized for laser beam splitting and coupling. After entering the optical path module, the laser is frequency-shifted by an AOM, and the optical path is extended via a set of reflecting mirrors. In addition, the zero-order beam is blocked by laser-absorbing black paper. Furthermore, all mechanical structures inside the optical path enclosure are treated with blackening to suppress laser leakage from various components to the greatest extent. With this configuration, the beam-splitting loss of the laser can be controlled within 15%.

**Figure 3:**
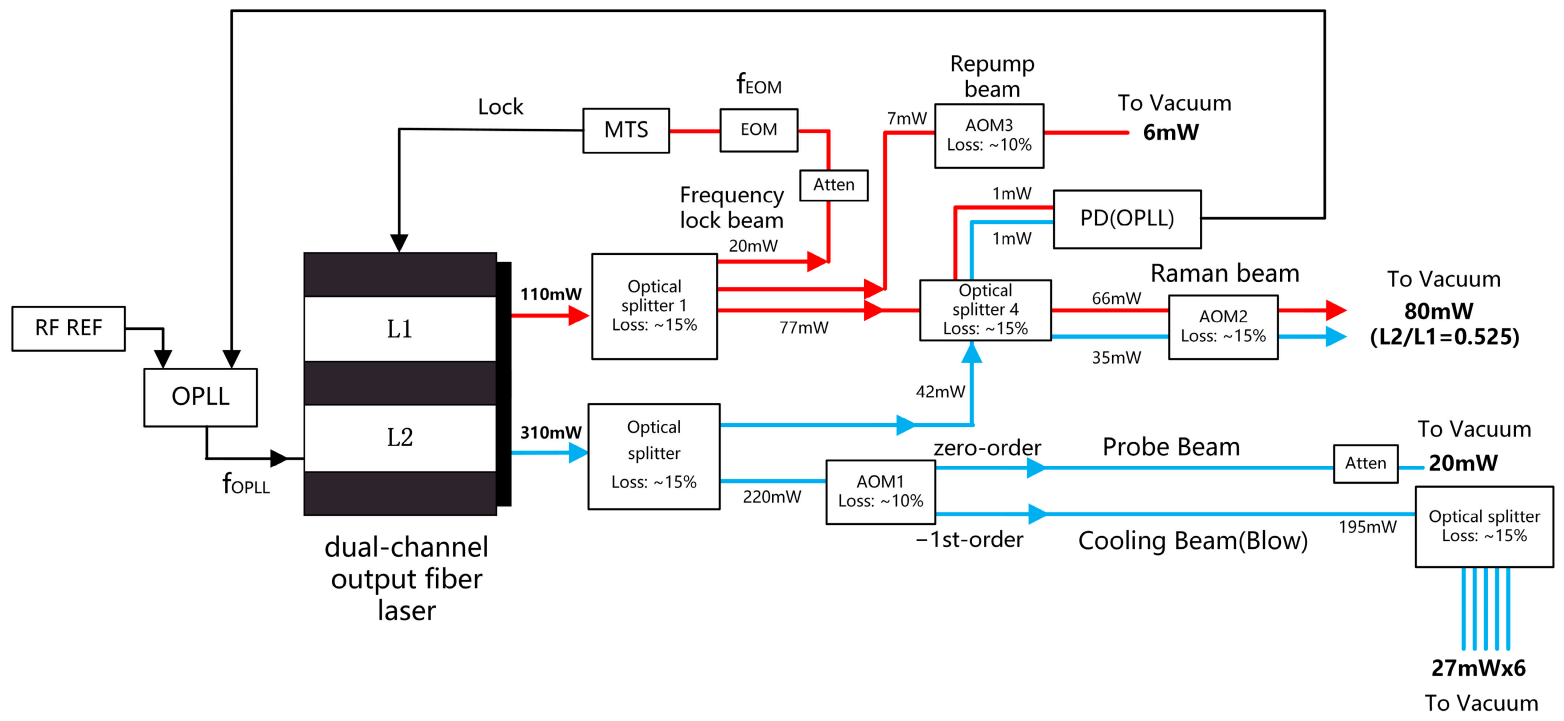
Design drawing of optical path power transmission scheme.

The theoretically calculated laser power transmission distribution is illustrated in Figure 3. With output power of 110 mW for the L1 laser and 310 mW for the L2 laser, the transmitted power after maximum theoretical optical loss can fully satisfy the power requirements of all lasers throughout the atomic interferometry process.

**Figure 4:**
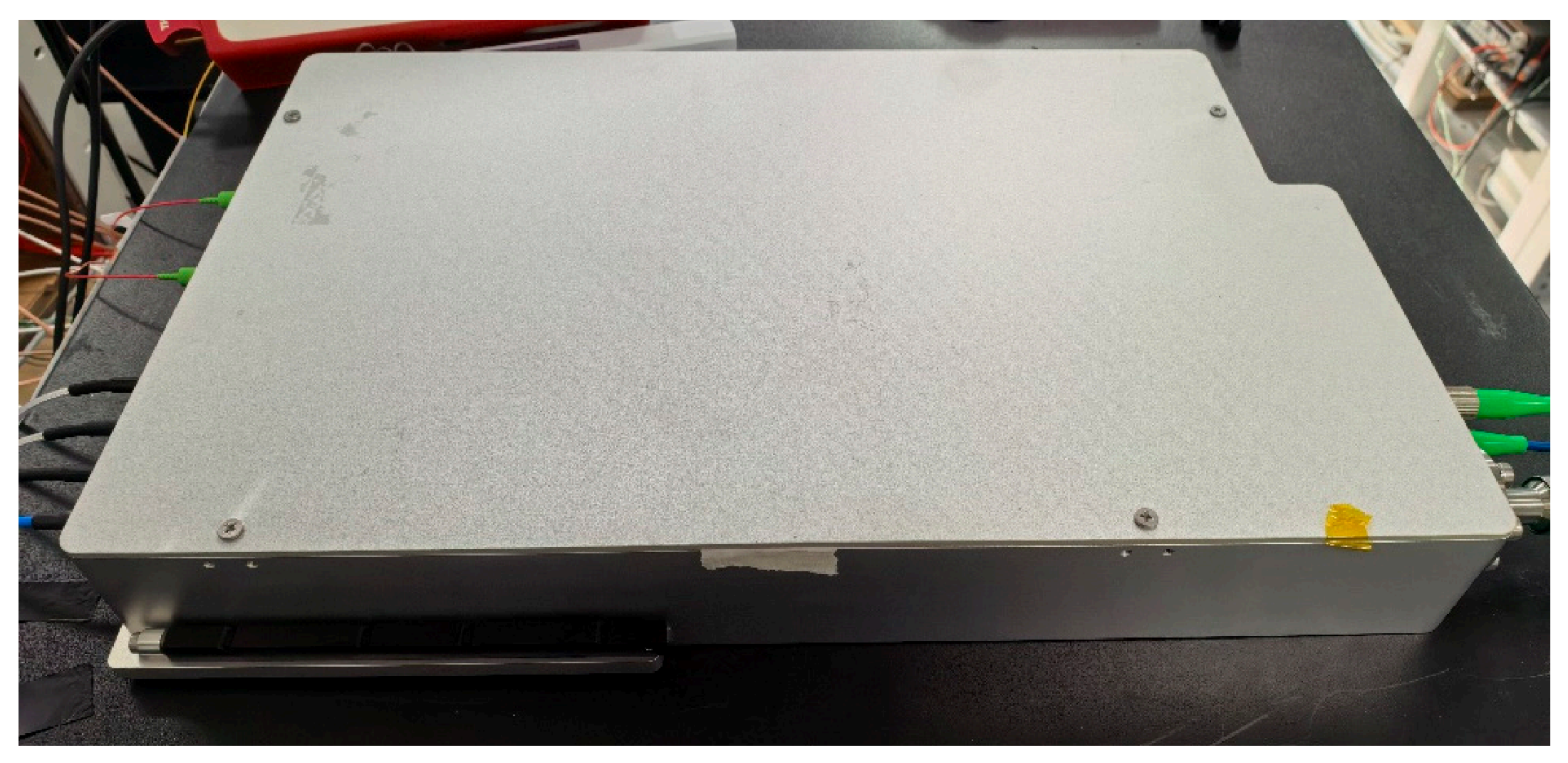
Physical photograph of the optical path system.

The physical image of the finally constructed and adjusted optical path is shown in Figure 4. All optical paths are integrated into a metal box with dimensions of 345 × 215 × 48 mm. The metal box is finally installed in a chassis for measurement. The external interfaces of the integrated optical path include the main laser and slave laser inputs, laser outputs, laser power monitoring ports, and RF drive interfaces. Installation can be completed rapidly for mobile field tests.

**Figure 5:**
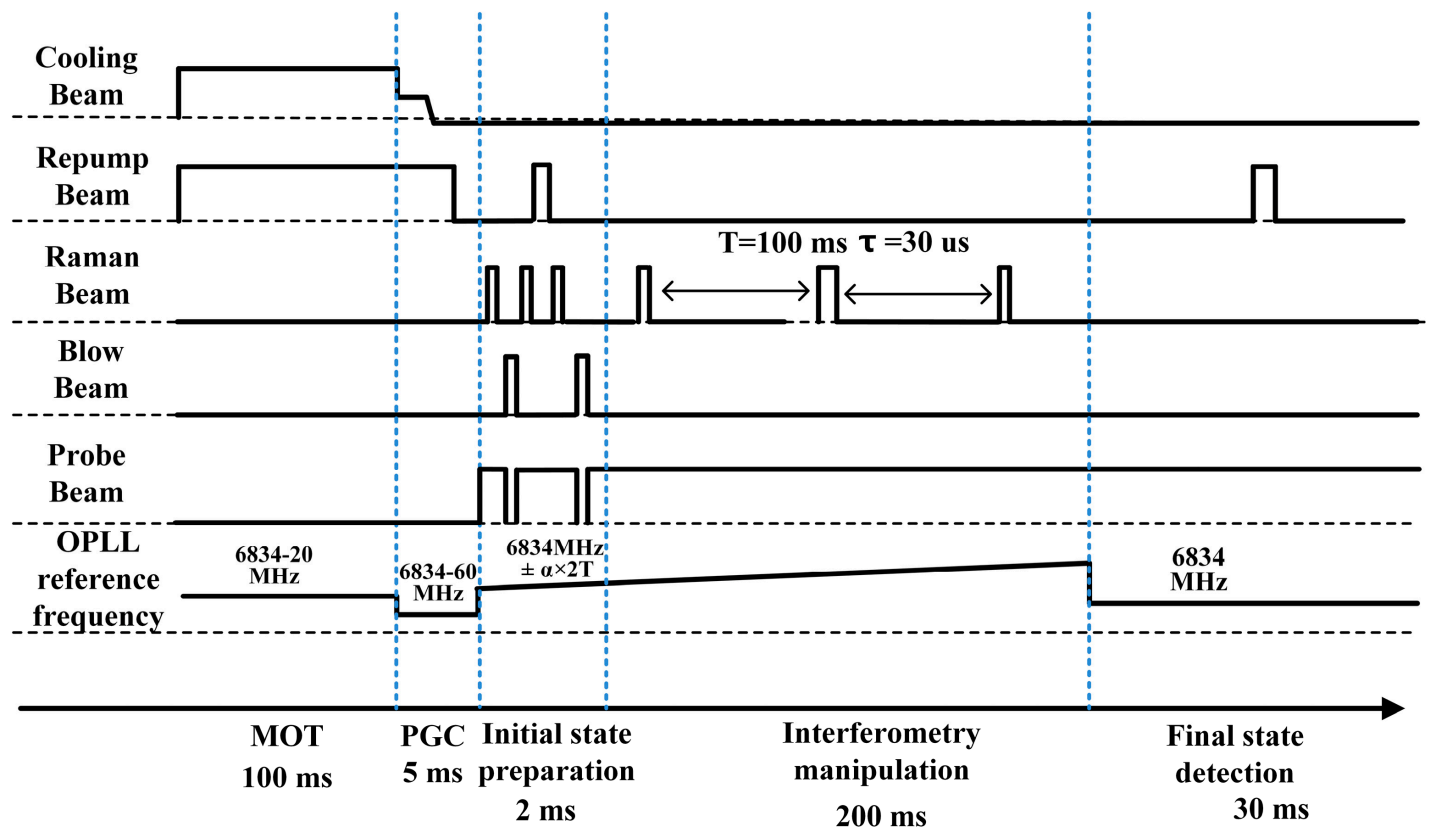
Timing sequence diagram of atomic interferometry gravity gradient experiment based on dual frequency-shifting optical path.

Based on the above design, the timing sequence of the entire atomic interferometry gravity gradient measurement is shown in the following Figure 5.

First, the RF for the cooling beam and repump beam is switched on. At this moment, the reference signal source of the optical phase-locked loop (OPLL) outputs a frequency of 6834-20 MHz for atom trapping. After a 100 ms loading period, the OPLL reference source changes its output frequency to 6834-60 MHz. Meanwhile, the RF power of the cooling beam is reduced to perform polarization gradient cooling (PGC), which lasts for 5 ms. The repump beam RF is turned off within 1 ms after the cooling beam is completely shut down.

Subsequently, initial state preparation is carried out. The frequency of the OPLL reference source jumps back to 6834 MHz, and chirp scanning starts. The RF switching sequence is: Raman π pulse → blow beam→ Raman π pulse → repump beam → Raman π pulse → blow beam, with the whole process lasting 2 ms.

Afterwards, the system enters the interference interaction time. The Raman beam is sequentially turned on to generate π/2 − π − π/2 pulses with an interval of 100 ms between each pulse. Upon completion of the interference, the OPLL reference source jumps to 6834 MHz after a 5 ms delay. At this point, the zero-order beam of the cooling beam acts as the horizontal probe beam for final-state detection, and the interference signal is acquired, completing one measurement cycle.

### 2.3. High-Isolation-Ratio RF Circuit System

In atomic interferometric gravity gradient experiments, residual leakage of cooling, repump, and Raman lasers induces undesired laser–atom coupling during the free evolution stage of atoms, leading to random atomic frequency shifts. Meanwhile, leaked photons trigger atomic spontaneous emission and disrupt the coherent superposition state of atomic matter waves, which reduces the fringe contrast and signal-to-noise ratio of interference patterns and ultimately limits the measurement sensitivity. Near-resonant optical leakage additionally generates radiative pressure, which causes atomic cloud diffusion and density attenuation during atomic free fall. This reduces the number of atoms participating in interference and further degrades the detected signal intensity.

To address the above issues, mechanical optical switches are conventionally adopted in traditional atomic interferometric laser systems for time-sequence laser gating, suppressing off-state optical leakage during non-working periods. By physically blocking the optical path, mechanical switches reduce residual leakage of Raman, cooling, and probe lasers during atomic interferometric evolution and avoid persistent stray-laser disturbance on atomic clouds. Nevertheless, mechanical switches have inherent drawbacks, including limited response speed, finite service life, and inevitable mechanical wear and positioning drift during long-term operation. Such defects severely degrade the stability and reliability of mobile measurement systems that require high-frequency and long-duration continuous operation. Furthermore, the introduction of mechanical switches increases structural complexity and assembly difficulty, hindering the miniaturization and integration of optical systems, which conflicts with the requirements of mobile atomic interferometric gravity gradiometers for lightweight design and high environmental adaptability [19].

In the system designed in this study, since the probe beam operates at the vertical position of cavity detection, optical leakage can be neglected. For the other lasers, including the cooling beam (blow beam), repump beam, and Raman beam, stray laser suppression is required. All these lasers are generated by RF-driven acousto-optic modulators (AOMs). The optical leakage of AOMs mainly consists of two components: the zero-order leakage and the diffracted-order leakage.

The zero-order AOM leakage arises from two sources. If it originates from a fiber-coupled zero-order laser, it is generally stable. However, if it comes from a device with a stray laser, it may fluctuate significantly under the influence of environmental vibrations. To suppress such zero-order leakage, we typically optimize the optical path structure and improve the selection of optical components to reduce the stray laser.

Fluctuations in the diffracted AOM leakage are mainly caused by variations in the driving radio-frequency signal. In conventional atomic interferometric optical systems, mechanical optical switches are often used to completely block diffracted leakage. To solve this problem, this study proposes a high-isolation-ratio RF driving control method to suppress diffracted optical leakage without using mechanical optical switches. This approach not only avoids vibration interference, response delay, and structural redundancy caused by mechanical switches but also provides reliable support for the miniaturization, integration, and long-term stable operation of the system.

#### 2.3.1. Analysis of Laser Isolation Ratio Requirements in Atomic Interferometry Experiments

First, the influence of cooling beam leakage on atomic interference is analyzed. From the end of the polarization gradient cooling (PGC) to the state-preparation stage, the stray cooling beam induces stimulated transitions of atoms between the energy levels F = 2 and F′ = 3, while transitions corresponding to other energy levels can be neglected, which leads to the heating of the atomic ensemble. Assuming that the initial temperature of the atomic cloud is 4 μK, a single stimulated transition can increase the atomic cloud temperature to approximately 4.3 μK. Accordingly, to suppress the adverse effects of cooling beam leakage, the number of stimulated transitions of a single atom during the laser–atom interaction process should be controlled to less than 1, so that the resultant heating of the atomic cloud can be ignored. During the state-selection stage before the three-pulse atomic interference operation, cooling beam leakage also causes dominant F = 2 to F′ = 3 stimulated transitions of atoms, with all other energy level transitions negligible. Even a single stimulated transition occurring in the state-selection process can raise the atomic cloud temperature by approximately 0.35 μK, which seriously undermines the subsequent manipulation of atomic interference. In the interference stage after the first π/2 interference pulse, the leaked cooling beam can still trigger F = 2 to F′ = 3 stimulated atomic transitions, and other transition channels remain negligible. Any occurrence of such stimulated transitions will destroy the atomic interference process, and the excited atoms will form thermal background noise for atomic interference fringes, thereby deteriorating the interference contrast. For all the above-mentioned physical processes, the average number of stimulated transitions per atom should be far less than 1 (e.g., 0.01 or even 0.001) to ensure that the leakage-induced disturbance is negligible. The transition probability of atoms under the action of the laser field is expressed as follows:

(1) $$W_{12}=\frac{\Gamma }{2}\cdot \frac{s}{1+s+{\left(\frac{2\delta }{\Gamma }\right)}^{2}}$$

where Γ is the atomic spontaneous emission rate, $\Gamma =36.1\times 10^{6}\;s^{-1}$, δ is the frequency detuning, and s is the saturation factor. $s=\frac{I}{I_{s}},\;I_{s}=1.67\;\mathrm{mW}/{\mathrm{cm}}^{2}$.

In this section, the cooling beam leakage level is quantified under two constraint conditions where the average atomic transition number is set to 0.01 and 0.001. According to the frequency offset configuration of the cooling beam in the experiment, the zero-order cooling beam adopts a relative detuning of δ = +133 MHz. After the polarization gradient cooling (PGC) process, the atomic cloud enters the free-fall state. The Gaussian radius ω is defined as the effective boundary of the cooling beam field, and the time required for the atomic cloud to completely exit the cooling beam coverage area is calculated. In the experimental system, a lens with a focal length of 100 mm is utilized for cooling beam collimation. The laser beam is expanded and then injected into the vacuum cavity in a standard Gaussian mode, with a collimated Gaussian beam radius of 9.4 mm. The average power density of the Gaussian beam can be described by the following formula:

(2) $$I_{0}=\frac{P}{\pi \omega^{2}}$$

The average saturation power is calculated to be *P_s_* = 4.6 mW with a beam radius of ω=9.4 mm and a saturation intensity of $I_{0}=I_{s}=1.67\;\mathrm{mW}/{\mathrm{cm}}^{2}$.

The free-fall time of atoms passing through the cooling beam Gaussian region with an average power of *P_s_* is approximately t = 43 ms. To simplify the calculation, the interaction time between atoms and the leaked cooling beam is set to 43 ms in the experiment, and the influence of atomic population in the F = 1 ground state is neglected. The transition number of atoms during the free-fall flight out of the cooling beam coverage region is expressed as $n=t\times W_{12}$. When the transition number of a single atom is 0.01 during the whole process, the calculated saturation parameter s is 6.3 × 10. Combined with the calculation formula of the saturation parameter, the allowable leakage laser power is determined to be 2.9 nW. For a total cooling beam power of 200 mW, the required isolation ratio reaches 80 dB. Similarly, when the atomic transition number is reduced to 0.001, the allowable leakage laser power is 0.2 nW, corresponding to a required isolation ratio of 91 dB.

This section further analyzes the influence of repump beam leakage on atomic interference. During the state-selection stage before the three-pulse atomic interference sequence, the residual repump beam induces stimulated transitions of atoms from the F = 1 energy level to the F′ = 2 energy level, while transitions of other energy levels are negligible, which completely destroys the state-selection process. In the interference stage after the excitation of the first π/2 interference pulse, repump beam leakage still dominates the F = 1→F′ = 2 stimulated transition behavior with negligible transition channels of other energy levels. Any occurrence of such stimulated transitions will disrupt the coherent evolution of atomic interference. The excited atoms serve as thermal background atoms for interference fringes and significantly degrade the interference contrast. To eliminate the above adverse disturbances, the average number of single-atom stimulated transitions in this stage should be far less than 1, and the interference error can be ignored only when the transition number is controlled to 0.01 or even 0.001.

The repump beam resonates with atoms after a 400 MHz frequency shift, and the interference induced by the atomic F = 2 ground-state population is neglected in the analysis. By adopting the same analytical method applied to cooling beam leakage, the requirements for repump beam diffracted laser leakage power and the corresponding optical isolation ratio under different transition probabilities are derived. When the average single-atom transition number is 0.01, the actual effective power of the repump beam is doubled due to reflection from the bottom mirror. In this case, the maximum allowable leakage power is restricted to 0.005 nW. Given an output repump beam power of 6 mW in the theoretical design, the required optical isolation ratio is 91 dB. Correspondingly, the isolation ratio needs to be improved to 103 dB when the atomic transition number is reduced to 0.001.

Finally, the influence of Raman beam leakage on atomic interference is analyzed from two aspects, including single-photon transition and two-photon transition effects. For the single-photon transition analysis, the analytical framework of cooling beam leakage is adopted for reference. This work only considers the leakage effect induced by the cooling beam component of the Raman beam. The repump beam component in the Raman beam exhibits a larger frequency detuning, and its single-photon transition effect is negligible compared with that of the cooling beam component. The diffracted Raman beam has a fixed relative detuning of +400 MHz. Following the identical analytical method for cooling beam leakage, the required optical isolation performance is quantified. Given an output power of 40 mW for the cooling beam component of the Raman beam, the required optical isolation ratio is 57 dB when the average single-atom transition number is 0.01 throughout the entire experimental process, and the required isolation ratio increases to 67 dB for a transition number of 0.001.

Furthermore, the two-photon transition effect induced by Raman beam leakage is investigated. A residual leaked Raman beam can trigger two-photon transitions, introducing amplitude noise and fringe fluctuations, which deteriorate the contrast of atomic interference fringes. The Raman beam propagates along the same direction as the atomic free-fall motion, so the leaked laser interacts with the atomic ensemble during the entire interference measurement period, with an effective interaction duration of 200 ms in the experiment. For a Raman beam power of 80 mW, the π-pulse duration is set to τ = 15 μs, which corresponds to a unit atomic transition probability (n = 1) under pulsed Raman beam excitation. Based on the calculation formula of (τ × n)/t, the allowable leakage power and corresponding isolation ratio are determined. To restrict the atomic transition probability to 0.01, the maximum tolerable Raman beam leakage power is 40 nW, requiring an optical isolation ratio of 63 dB. For a lower transition probability of 0.001, the allowable leakage power is reduced to 4 nW, and the required optical isolation ratio reaches 73 dB.

#### 2.3.2. RF Scheme Design with Ultra-High Isolation Ratio

**Figure 6:**
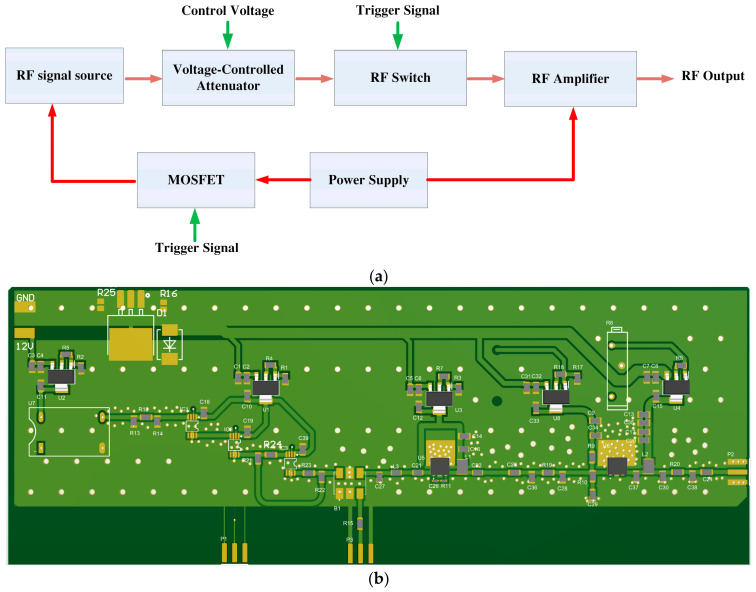
Schematic of the RF driver circuit. (**a**) RF circuit scheme design. (**b**) RF circuit simulation model.

This RF drive system is composed of an RF signal source, voltage-controlled attenuator, RF switch, RF amplifier, and MOSFET, forming a complete signal path, which is divided into an RF signal transmission link and a control part. The RF signal source generates a reference RF signal and transmits it to the voltage-controlled attenuator, realizing continuous adjustment of output power through externally input control voltage; the RF signal is then transmitted to the RF switch, and the on-off of the path is controlled by an external trigger signal to complete time-sequence gating of the RF signal. The RF signal gated by the switch is sent to the RF amplifier for power amplification and finally outputs a standard RF signal. The power supply unit uniformly provides working power for the RF amplifier and the MOSFET. The MOSFET receives independent trigger signals and serves as the power switch of the RF signal source to control the on-off of the oscillation source. Compared with the traditional scheme that only relies on RF switches for isolation, this design adopts a MOSFET to cut off the power supply loop of the RF signal source, which directly turns off the RF oscillation source from the signal source, thoroughly eliminating the background RF leakage when the oscillator is off. Cooperating with the path isolation of the rear-stage RF switch, it significantly improves the overall RF isolation ratio of the system, achieves superior off-state RF suppression effect, effectively avoids signal crosstalk and stray leakage, and guarantees the purity of output RF signals and the control accuracy of the system. The overall scheme design is shown in Figure 6. In the design of RF circuits, the core commercial components selected are as follows: the MOSFET model CRSS037N10N from China Resources Microelectronics Limited (Suzhou, China); the diode model 1N4001M1 from Shenzhen JSMSEMI Semiconductor Co., Ltd. (Shenzhen, China); the high-speed RF switch model SIS084SP3 from Chengdu SiCore Semiconductor Co., Ltd. (Chengdu, China); and a custom frequency crystal oscillator provided by Shenzhen Ruijing Electronics Co., Ltd. (Shenzhen, China).

**Figure 7:**
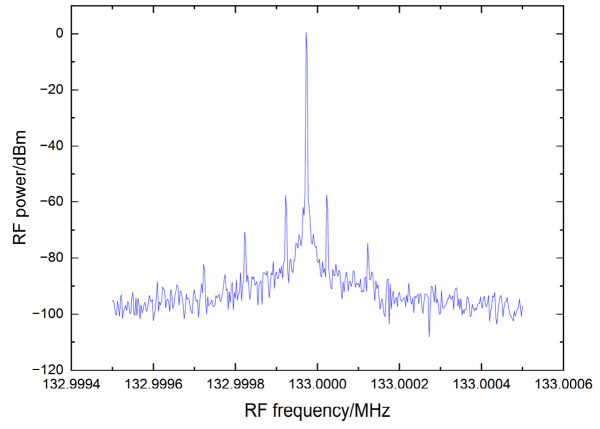
RF output power of 133 MHz, reaching 30.46 dBm (with 30 dBm attenuation added to prevent damage to the spectrum analyzer from excessive power, the spectrum analyzer is set to RBW 1 Hz, VBW 1 Hz, and RF attenuation 30 dB).

Taking the 133 MHz RF signal required for the experiment as an example, the RF power can reach 30.46 dBm when output using the designed circuit. The measured output power results are shown in Figure 7.

**Figure 8:**
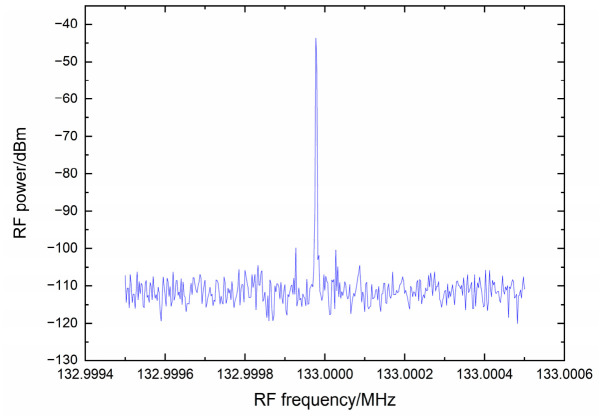
Residual 133 MHz RF power after turning off the RF switch and control voltage without using MOSFETs. (The spectrum analyzer is set to RBW 1 Hz, VBW 1 Hz, and RF attenuation 30 dB).

In the RF circuit without MOSFETs as isolation devices, after turning off the RF switch and control voltage, the isolation ratio can only reach 74.17 dB. The measured residual RF power is shown in Figure 8.

**Figure 9:**
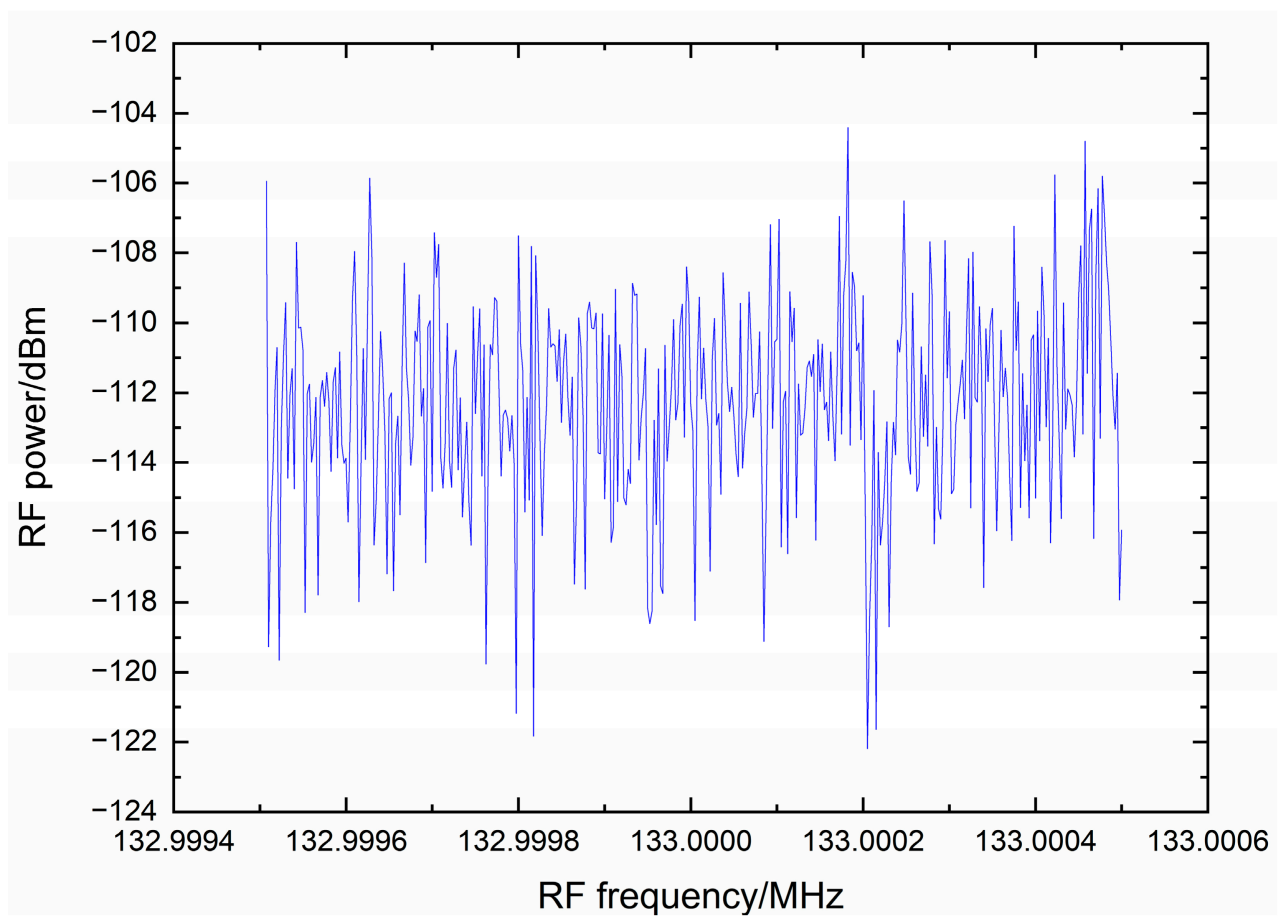
Measured residual RF power after turning off the MOSFET (the spectrum analyzer is set to RBW 1 Hz, VBW 1 Hz, and RF attenuation 30 dB).

After turning off the MOSFET, the residual RF power is close to the noise floor of the spectrum analyzer and is nearly undetectable. When the load is removed, the instrument noise floor is consistent with the residual RF power measured with the MOSFET turned off, as shown in Figure 9.

**Figure 10:**
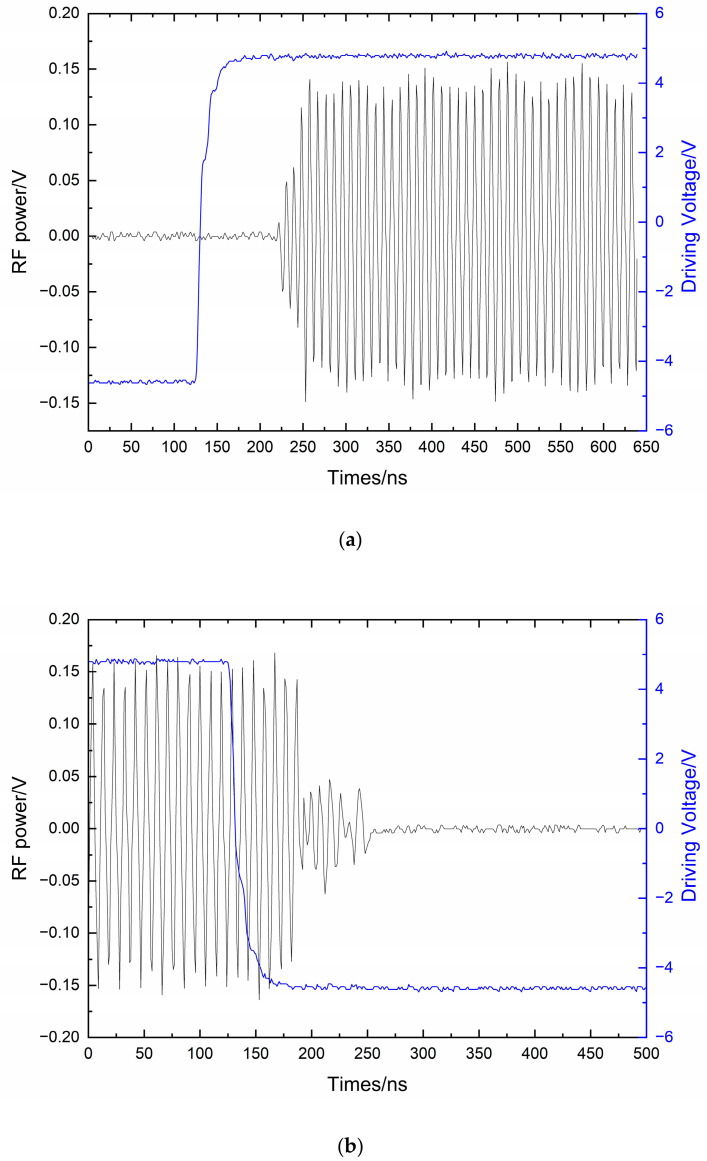
Turn-on and turn-off times of the RF circuit using MOSFETs as isolation devices. (**a**) The turn-on time of the RF signal reaches approximately 125 ns (the blue line represents the control signal, and the black line represents the RF signal). (**b**) The turn-off time of the RF signal is only 100 ns (the blue line represents the control signal, and the black line represents the RF signal).

However, the only risk of using MOSFETs as isolation devices is a potential increase in the turn-on delay of the RF circuit. By optimizing the MOSFET control voltage and switching timing, the delay in turning RF signals on and off using MOSFETs is shown in Figure 10.

**Figure 11:**
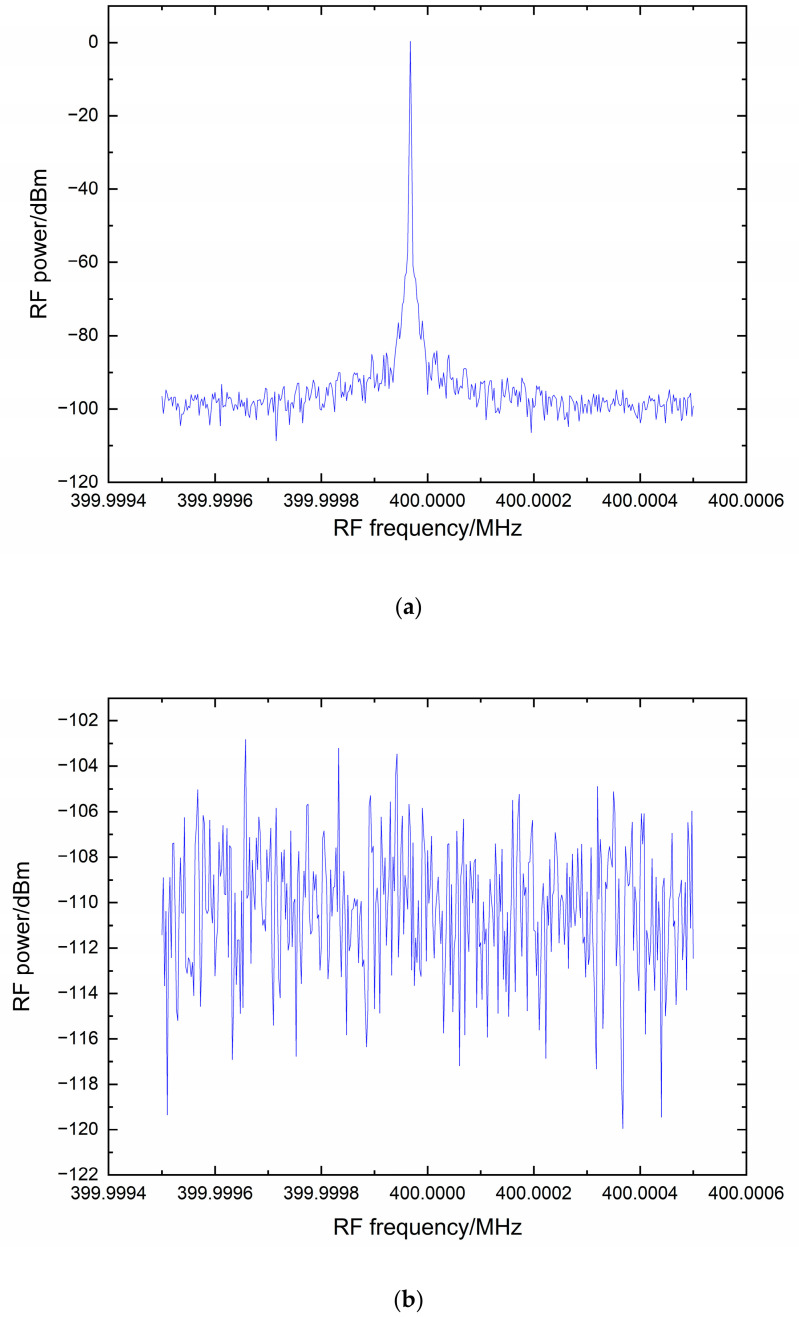
Measured output power and residual power of the 400 MHz RF signal after passing through the high-isolation RF circuit. (**a**) Measured 400 MHz RF power (with 30 dBm attenuation added to prevent damage to the spectrum analyzer from excessive power). (**b**) Measured 400 MHz RF power after high-isolation RF switching (the spectrum analyzer is set to RBW 1 Hz, VBW 1 Hz, and RF attenuation 30 dB).

The measured isolation ratio of the 400 MHz RF driver for the repump and Raman beams is shown in Figure 11.

To verify the effectiveness of the high-isolation radio-frequency circuit system in suppressing laser leakage, we measured the laser power using a Thorlabs SC120 power meter (Thorlabs, Inc., Newton, NJ, USA) with an S120-APC fiber flange (Thorlabs, Inc., Newton, NJ, USA) and a PM400 main unit (Thorlabs, Inc., Newton, NJ, USA). When the laser was completely turned off, the power meter reading was 1.04 nW. After the laser was turned on, the total power at its output ports was 105 mW for the first channel and 300 mW for the second channel. The laser power finally delivered to the output of the optical system was measured as follows: the cooling beam power was 203 mW, and the power meter showed 2.46 nW when the radio-frequency electronic control system was turned off; the repump beam power was 8.5 mW, with a reading of 1.05 nW under the same off condition; the single-channel power of the horizontal probe beam after 1 × 2 splitting was 18 mW, whose extinction ratio was not considered since it is the zero-order laser of the cooling beam and has no additional influence on the measurement; and the Raman beam power was 79 mW, with the power meter indicating 3 nW when the radio-frequency electronic control system was switched off.

Regarding the results of electrical power consumption control, we compared the traditional optical path and radio-frequency (RF) system employed for gravity gradient measurements in our laboratory, in which the cooling beam utilizes a 107 MHz double-pass optical path, the repump beam uses a 230 MHz double-pass optical path, the probe beam uses a 197 MHz detuned optical path, the blow beam uses a 197 MHz detuned optical path, and the Raman beam uses a 200 MHz detuned optical path. The power consumption of the laser and the electronic control chassis under normal experimental operation was measured separately using an HP-8713 power meter (Shenzhen Hongpin Electronic Technology Co., Ltd., Shenzhen, China). For the laser, when using the same type of laser device, the traditional optical path requires both laser channels to provide an output power of 1 W, resulting in an electrical power consumption of 200.1 kWh, whereas the electrical power consumption of the laser in this study is 137.3 kWh.

For the RF circuit, as it is connected to the chassis via a VPX interface and benefits from the simplified RF architecture, the power consumption of multiple modules, including the power supply and heat dissipation, has been effectively reduced. The overall power consumption of the chassis in the traditional scheme is 470.8 kWh, while that of the chassis adopted in this study is 253.3 kWh.

## 3. Results

Based on the complete laser optical path system established in this study, we performed a mobile atomic interferometry gravity gradient measurement experiment in the field. The field measurement area is located in a Gobi mining region, characterized by an open and unobstructed site with dry surface conditions and no significant environmental interference. The ambient temperature during outdoor operation ranged from 13 to 28 °C, with a relative humidity of 33%. After transporting the mobile atomic interferometry gravity gradiometer to the field site, multi-point static measurements were carried out using a point-by-point measurement method.

**Figure 12:**
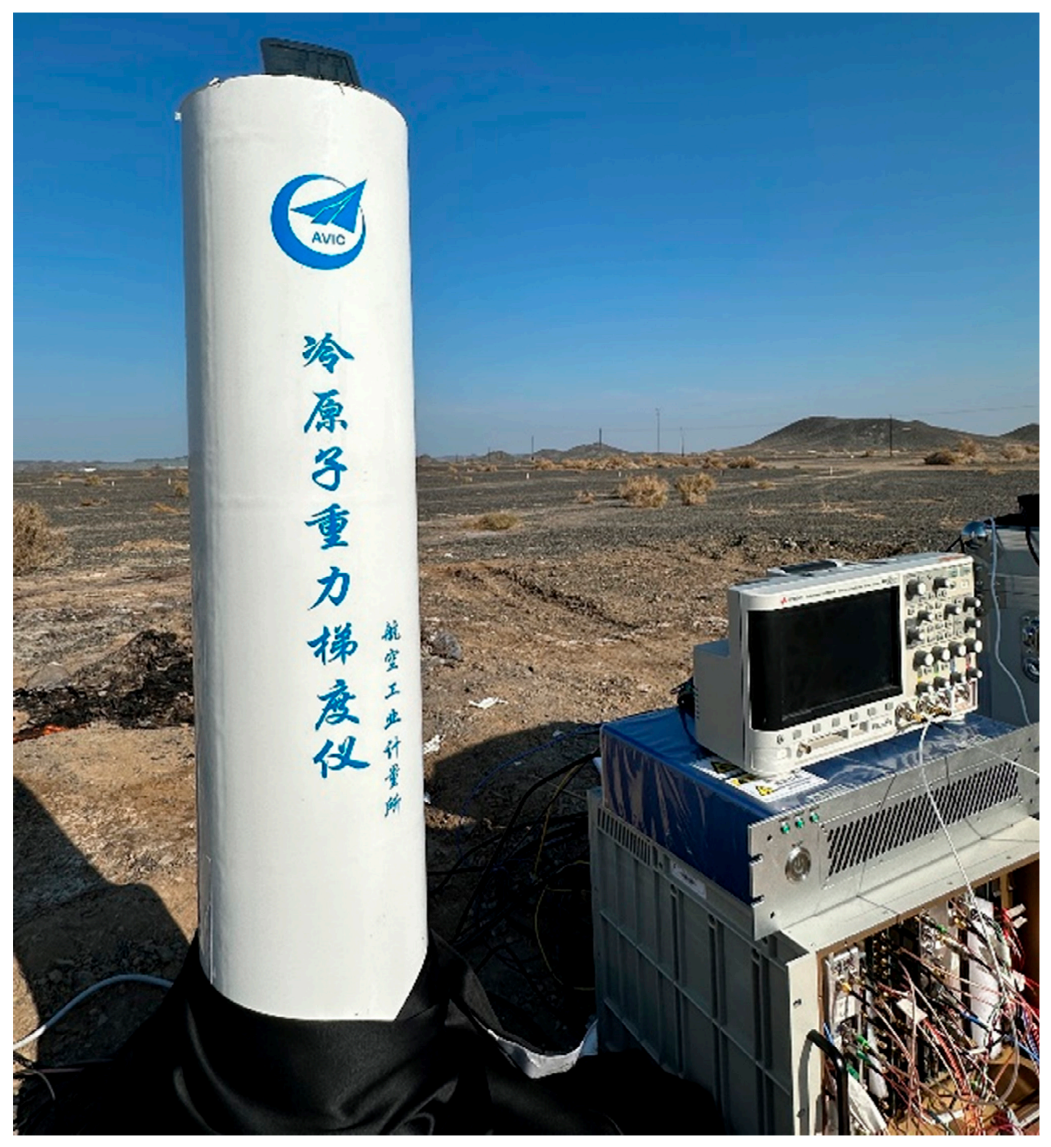
Field test of the atomic interferometry gravity gradiometer with the optical system developed in this study. (The Chinese text printed on the instrument: “冷原子重力梯度仪” denotes atom gravity gradiometer; “航空工业计量所” denotes Changcheng Institute of Metrology and Measurement).

First, the instrument was set up, leveled, and stabilized at the first pre-determined measurement point, followed by static gravity gradient data acquisition and testing. Upon completion of measurements at that point, the instrument was safely transferred to the next measurement site. The photograph of the field measurement site is shown in Figure 12.

**Figure 13:**
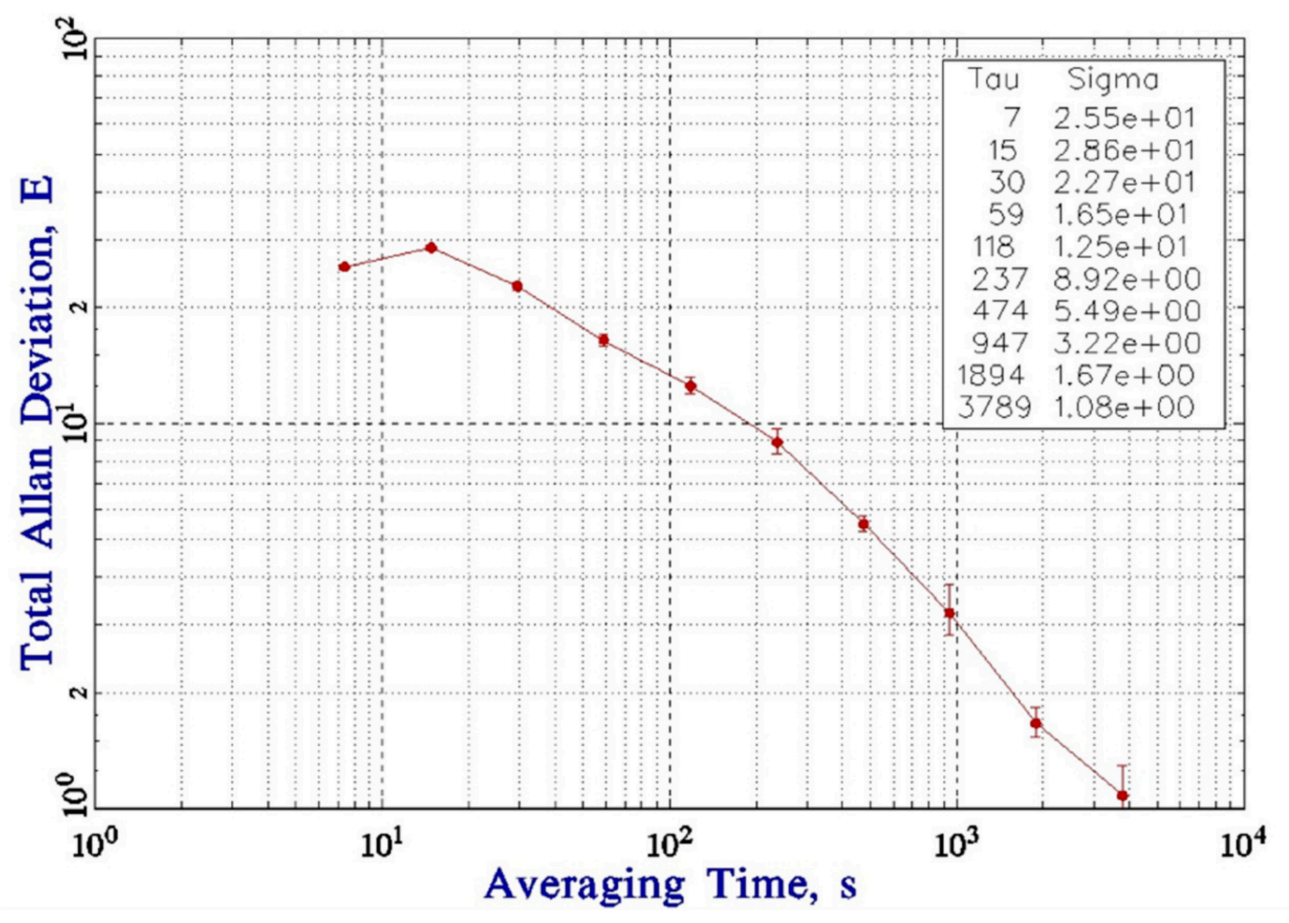
Long-term stability of the gravity gradient measurement.

The baseline of the whole atomic interferometric gravity gradiometer is 0.5 m, with an interference time of 100 ms, and it takes 7.5 s to acquire a single gravity gradient value. To evaluate the long-term stability, the Allan variance integration method is adopted to assess the long-term stability of atomic interferometric gravity gradient measurement results. Within an integration time of 3789 s, the long-term stability of gravity gradient measurement reaches 1.08 E (the results are shown in Figure 13). Meanwhile, we systematically analyze and evaluate the influences of major error sources including the Coriolis force, baseline length, and single-photon frequency shift. Finally, the combined standard uncertainty of the measured results is determined to be 26.3 E.

## 4. Discussion

To meet the engineering application requirements of atomic interferometry gravity gradient measurement, this study develops a highly integrated, low-power laser system. The proposed scheme optimizes the laser frequency-shifting optical path for atomic interferometry gravity gradient measurement. By adopting only two frequency shifts, 133 MHz and 400 MHz, it fully satisfies all laser operating requirements for atomic interferometry gravity gradient measurement. Compared with traditional laboratory-based schemes, the number of required acousto-optic modulators (AOMs) is reduced by two, and the complexity of the optical path is significantly simplified.

In terms of power consumption, this study constructs a dual-channel fiber laser output scheme, which matches the laser power specifications of the measurement system with input power of 105 mW and 300 mW. This configuration effectively reduces the loss of optical components under long-term operation and provides favorable protection for the optical path system. The electrical power consumption of the laser system and the electronic control chassis is reduced by 62.8 kWh and 217.5 kWh, respectively, compared with the traditional scheme, which guarantees long-term field measurements.

This study further analyzes the laser leakage issue in atomic interferometry gravity gradient measurement, specifies the isolation ratio technical indicators for laser leakage in each channel, and designs a high-isolation RF driving module accordingly. With this driving module, the system eliminates the hardware dependence on traditional mechanical laser shutters and realizes precise laser leakage suppression and laser manipulation without mechanical shutters. This design significantly simplifies the hardware architecture and debugging procedures of existing laser systems for atomic interferometry gravity gradient measurement, effectively reduces the costs of system construction, operation, and maintenance, and improves the stability and environmental adaptability of the measurement system, exhibiting excellent engineering application performance in mobile field test scenarios.

Atomic interferometry gravity gradient measurements based on this integrated laser system achieve a long-term stability of 1.08 E, which is comparable to reported results of state-of-the-art mobile static measurements and atomic interferometry gravity gradient studies. This fully verifies the feasibility of applying the integrated and lightweight optical path scheme to high-precision atomic interferometry measurement. This study can provide reliable technical support and reference for the practical application and engineering implementation of atomic interferometry gravity gradiometers, as well as their popularization in vehicle-mounted and mobile scenarios.

## Data Availability

The raw data supporting the conclusions of this article will be made available by the authors on request.

## Abbreviations

The following abbreviations are used in this study:

PGC	Polarization gradient cooling
MOT	Magneto-optical trap
AOM	Acousto−optic modulator
MTS	Modulation transfer spectroscopy
